# Metacognitive Therapy in Cardiac Rehabilitation: Staff Barriers and Enablers to Training in an Evidence‐Based Psychological Treatment for Anxiety and Depression

**DOI:** 10.1161/JAHA.125.044672

**Published:** 2026-07-17

**Authors:** Adrian Wells, Andrew Belcher, David Reeves, Patrick Doherty, Paul Wilson, Lora Capobianco

**Affiliations:** ^1^ Division of Psychology and Mental Health, School of Psychological Sciences, Faculty of Biology, Medicine and Health The University of Manchester United Kingdom; ^2^ Research and Innovation Greater Manchester Mental Health NHS Foundation Trust, National Health Service Foundation Manchester United Kingdom; ^3^ NIHR School for Primary Care Research, Williamson Building, Manchester Academic Health Science Centre The University of Manchester United Kingdom; ^4^ Jean McFarlane Building, Faculty of Biology Medicine and Health, Centre for Biostatistics, School of Health Sciences The University of Manchester United Kingdom; ^5^ Division of Population Health, Health Services Research and Primary Care, School of Health Sciences University of Manchester United Kingdom; ^6^ Department of Health Sciences University of York United Kingdom

**Keywords:** cardiac rehabilitation, cardiovascular disease, implementation, mental health, metacognitive therapy, Health Services

## Abstract

**Background:**

This study was designed to explore cardiac rehabilitation (CR) practitioners' experiences and perceptions of group metacognitive therapy training, identifying the facilitators and barriers encountered.

**Methods:**

A nested qualitative study using reflexive thematic analysis was conducted as part of PATHWAY‐Beacons (National Institute for Health and Care Research 202956), a mixed‐methods study evaluating the implementation of group metacognitive therapy as part of routine care in CR services. Practitioners from 6 CR services in England enrolled in interviews at 3 stages: before training, after training, and after delivering all group sessions.

**Results:**

Nine practitioners participated in the study, with 7 completing all 4 interviews, 1 completing 2 interviews (pre‐ and posttraining), and another completing just 1 (pretraining). Key enablers for effective training emerged in 3 subthemes: training delivery style, essential training components, and the online training format. These aspects contributed positively to the practitioners' training experiences and improved their understanding of group metacognitive therapy. Conversely, barriers included issues related to training structure, staff skepticism, and specific additional training needs.

**Conclusion:**

The findings support the positive value of training CR staff in group metacognitive therapy and offer important insights for enhancing the delivery and uptake of training within the National Health Service. Although the study findings represent views of a predominantly female sample, this reflects the composition of the current CR workforce.

**Nonstandard Abbreviations and Acronyms**
CR: cardiac rehabilitation
EBI: evidence‐based interventions
MCT: metacognitive therapy

**Research Perspectives**

**What Is New?**

- This is the first study to investigate the training experiences of cardiac rehabilitation practitioners in group metacognitive therapy.

**What Are the Clinical Implications?**

- Training in metacognitive therapy was positively received by cardiac rehabilitation practitioners, who have limited prior knowledge of complex evidence‐based psychological therapy, supporting the transferability of metacognitive therapy to cardiac rehabilitation settings.
- Within cardiac rehabilitation services, it would be advantageous for practitioners to engage in training in metacognitive therapy to enhance psychological knowledge and skills and support the unmet psychological needs of patients.

Cardiac rehabilitation (CR) is an evidence‐based intervention that has been clinically proven to be cost‐effective in enhancing physical, psychosocial, and health‐related quality of life outcomes for patients with cardiovascular disease.^1^, ^2^, ^3^, ^4^, ^5^ CR includes exercise training, health education, cardiovascular risk management, and psychological support.^6^

Anxiety and depression are common among patients with cardiovascular disease and are often associated with higher mortality rates, diminished quality of life, and lower attendance and adherence to CR programs.^7^, ^8^, ^9^, ^10^, ^11^, ^12^, ^13^, ^14^, ^15^, ^16^, ^17^, ^18^, ^19^ As such, effective psychological support is crucial in CR programs.^20^

UK and European guidelines from the British Association for Cardiovascular Prevention and Rehabilitation and the European Association of Preventive Cardiology emphasize the importance of psychosocial management as a core element of CR.^6^, ^21^, ^22^ This includes conducting psychosocial assessments, providing interventions throughout rehabilitation, measuring outcomes, and coordinating care with other health care providers.^6^, ^21^, ^22^

Despite the British Association for Cardiovascular Prevention and Rehabilitation guidelines, the availability of psychosocial support varies widely in CR programs in the United Kingdom. When psychological support is available, it typically involves stress management techniques (eg, relaxation and mindfulness) delivered by nurses, physiotherapists, or occupational therapists.^6^ The availability of expert mental health providers, such as psychologists, also varies widely; when available, they are often stretched across multiple physical health services.^23^ In view of such limitations, facilitating the delivery of psychological interventions by CR staff may help improve access to the support needed by patients and improve outcomes.

Although there are advantages to having CR practitioners provide psychological support, these professionals have received limited or no training in identifying and managing psychological problems, and they face time constraints due to the extensive curriculum of CR.^24^ Nevertheless, CR patients believe CR practitioners are well positioned to offer psychological support.^24^, ^25^ Patients report that CR staff's delivery of psychological interventions positively affects their experiences, creating a reassuring environment that encourages participation.^25^ Furthermore, CR staff also consider themselves ideally suited to provide psychological support to patients.^26^ Yet despite being well placed, CR practitioners often lack confidence in addressing this need, fear they may negatively affect patients’ well‐being, and often hesitate to discuss patients’ worries due to their own concerns.^26^

One solution to improving patients' mental health is to provide CR staff with the skills to deliver effective psychological interventions as part of the CR program. Research indicates that training nonpsychologists to deliver psychosocial treatments is both feasible and effective for long‐term physical health populations,^27^, ^28^, ^29^, ^30^, ^31^, ^32^, ^33^, ^34^ including those with diabetes,^27^, ^34^ cancer,^28,^ and cardiac conditions.^29^, ^30^ Trained professionals have effectively delivered psychological interventions such as motivational enhancement therapy, behavioral activation, and cognitive behavioral therapies across various medical settings.^27^, ^28^, ^29^, ^30^, ^31^, ^32^, ^33^, ^34^ However, the provision of specific interventions within CR has, until recently, been complicated by a shortage of high‐quality evidence on the effectiveness of psychological interventions in CR patients.^35^ In response to this situation, the National Institute for Health and Care Research ‐funded PATHWAY research program^36^, ^37^ evaluated the feasibility and efficacy of metacognitive therapy (MCT), an effective treatment in mental health clinics, when applied to CR patients. CR practitioners delivered MCT, and the combination of MCT + CR significantly reduced anxiety and depression at both 4‐month and 12‐month follow‐up, compared with CR alone.^37^ Additionally, it halved the rate of psychological deterioration among patients.

MCT may be well suited to CR as it is highly manualized, evidence based, and can be effectively delivered by trained CR staff. The results of the PATHWAY studies support future steps to evaluate group‐MCT implementation in CR services across England.^37^ However, it is increasingly recognized within implementation science that it is vital to understand the factors influencing the willingness and decision to adopt an intervention to support uptake in real‐world settings.^38^

Previous research on barriers and facilitators to adopting evidence‐based interventions (EBIs) among psychotherapists has highlighted that training‐related challenges are among the most significant issues.^39^ Barriers related to training encompass both objectively and subjectively experienced obstacles. Objective barriers include limited time and funding for training, a shortage of accessible local training opportunities, and insufficient ongoing support necessary for refining new skills, such as supervision and consultation. Subjective barriers involve a perceived lack of confidence in one's ability to perform the therapy successfully.^39^ If EBIs such as MCT are to be implemented and rolled out in CR services, evaluating the enablers and barriers to training the CR workforce is essential. The training experiences of CR practitioners in MCT have not been studied to date, and thus in the present study we aimed to explore and understand the facilitators and barriers to training within the context of a multisite National Health Service implementation study.

## METHODS

This nested qualitative study explored practitioners' experiences and perceptions of group‐MCT training and identified perceived facilitators and barriers to that training. It was conducted as part of PATHWAY‐Beacons (National Institute for Health and Care Research 202956), a mixed‐methods study evaluating the implementation of group MCT as part of routine care in 6 CR services across National Health Service services in the North West, Midlands, Yorkshire and Humber, and South West of England.^40^ The study has been reported in line with the Consolidated Criteria for Reporting Qualitative Research checklist,^41^ see Supplemental Material. Data are available upon reasonable request to the corresponding author.

### Ethical Approval

The study received ethical approval from the UK Health Research Authority and Health and Care Research Wales (REC reference: 22/HRA/2220).

### Participants

Study participants included CR health care professionals who received training in delivering group MCT. All trainees were invited to participate in semistructured interviews supported by an interview guide. CR practitioners were interviewed at 3‐time points: pretraining, pos‐training, and postdelivery (after delivering all groups). Data from all 3‐time points were used to capture practitioners' views over time and across their experiences.

Thirteen practitioners from 6 CR services in England were trained in MCT, with 9 practitioners agreeing to participate in an interview and providing written or audio‐recorded consent before their interviews. Seven staff members completed all 3 interviews, 1 completed 2 (pre‐ and posttraining) and 1 completed only 1 (pretraining). Table 1 presents an overview of the demographic characteristics of the interviewed CR practitioners. Practitioners were predominantly female (8 out of 9 individuals), with an average age of 49.1 years (range: 32–61) and an average experience of 13.8 years (range: 4–26) in cardiac rehabilitation.

**Table 1:** Demographic of MCT Practitioners

ID	Site	Age, y	Sex	Role	Y working in CR	Previous mental health training	Mental health training details
1	5	49	Male	Physiotherapist	10	Yes	Counseling
2	1	58	Female	Cardiac specialist nurse	15	Yes	Counseling
3	2	57	Female	Staff nurse	5	Yes	Mental health first aid
4	1	32	Female	Cardiac rehabilitation nurse	4	No	Not applicable
5	1	56	Female	Nurse consultant	26	No	Not applicable
6	2	29	Female	Exercise physiologist	6	No	Not applicable
7	5	61	Female	Cardiac rehabilitation nurse	20	Yes	Counseling
8	6	47	Female	Cardiac rehabilitation specialist nurse	17	No	Not applicable
9	3	53	Female	physiotherapist	22	Yes	Mental health first aid, British Association for Cardiovascular Prevention and Rehabilitation education courses, cognitive behavioral therapy course

CR indicates cardiac rehabilitation; and MCT, metacognitive therapy.

### Training in Group MCT

MCT is a brief psychological therapy grounded in the metacognitive model^42^ aimed at reducing common repetitive negative thinking patterns and behaviors that maintain anxiety and depression and shown to be effective in treating anxiety, depression, and trauma symptoms among patients undergoing CR. For further details of the intervention and its effectiveness, please refer to Wells et al.^37^

Thirteen CR staff members, a minimum of 2 from each service, participated in 3 half‐days of online training sessions led by A.W., the developer of MCT. The training program included didactic teaching, a review of MCT training videos, role‐playing exercises, an examination of the intervention manual, and a pilot therapy group. Practitioners received a comprehensive treatment manual to guide the delivery of group MCT. The first 2 training sessions were delivered before the pilot group was implemented, and the third training session occurred after the pilot group. The final training session reviewed pilot delivery, discussed challenges encountered in treatment, and offered further training.

### Data Collection

All interviews were conducted using a semistructured guide to facilitate a flexible exploration of individual experiences. The study authors developed an a priori interview guide with open‐ended questions and prompts to explore practitioners' experiences and perceptions of group MCT training while identifying perceived facilitators and barriers to that training. A.B. conducted all interviews online using a secure video conferencing system from September 2022 to July 2023. The interviews were recorded using an encrypted recorder. On average, each interview lasted 55 minutes, ranging from 30 to 82 minutes. The interviews were audio‐recorded and transcribed verbatim, with identifiable information removed by a third‐party organization. Although the interview guide structured the interviews, the conversations were open and fluid, incorporating prompts and clarifying questions as needed. Transcripts of interviews were generated by a third‐party service and checked for accuracy by A.B.

### Statistical Analysis

Data were analyzed using reflexive thematic analysis, as described by Braun and Clarke.^43^, ^44^ Reflexive thematic analysis involves 6 phases of qualitative analysis that aim to identify and interpret patterns of shared meaning across a data set.^45^ These 6 phases include (1) data familiarization, (2) coding, (3) generating initial themes, (4) reviewing themes, (5) defining and naming themes, and (6) refining and reporting themes. We adopted an essentialist approach, allowing for a straightforward understanding of motivations, experiences, and meanings. It is based on the assumption of a largely unidirectional relationship between meaning, experience, and language, suggesting that language reflects and facilitates our ability to articulate our meanings and experiences.^42^

The analysis was conducted using NVivo software. A.B. began by thoroughly familiarizing himself with the data set, reading and rereading the transcripts multiple times while annotating initial points of interest. Codes were developed throughout the data using an inductive‐semantic approach to minimize the influence of preexisting theories and research. Coding was an interactive process in which A.B. identified and labeled significant data features relevant to the study's aims. Initial themes were generated by combining codes with similar meanings. Subsequently, A.W. led a series of discussions with A.B. and L.C., during which the codes and themes were further developed, reviewed, refined, and examined for potential discrepancies. Finally, all authors reviewed the themes and reached a consensus. Participants did not provide feedback on the findings.

## RESULTS

**Figure 1:**
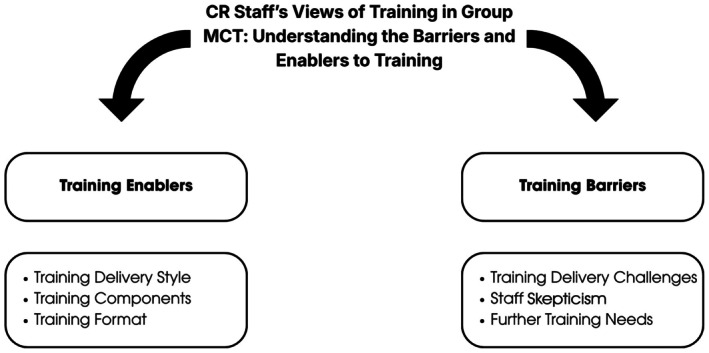
Overview of themes and subthemes. CR indicates cardiac rehabilitation; and MCT, metacognitive therapy.

Two main themes emerged from participants regarding their experience of training in group‐MCT (Figure1Figure). The first theme encompassed the enablers of training in group MCT, with the subthemes including training delivery style, essential training components, and the online training format. The second theme addressed the barriers to training in group MCT, with subthemes encompassing training delivery challenges, staff skepticism, and additional training needs.

### Theme 1: Training Enablers

The first theme included 3 subthemes related to training delivery style, essential training components, and the online training format. These factors facilitated a practitioner's training experience and were beneficial to developing their understanding of group‐MCT.

#### Training Delivery Style

CR practitioners felt that the training comprehensively covered MCT delivery. They appreciated the structured, chronological format, which allowed them to experience the entire group‐MCT intervention in a compact yet detailed training program. Despite being a complex psychological intervention, practitioners noted that they felt the training program was easy to follow.

“I really liked [how] we went through each session, and it was almost like … doing the whole 6 weeks with us in 2 days really, so it was a lot to get your head round, but I like the way it was delivered.” [ID 6]

#### Essential Training Components

The CR practitioners identified several key training elements for enhancing their knowledge, understanding, and application of group MCT. These elements included prerecorded training videos, the delivery of a pilot group, and the subsequent training session following the pilot group.

The prerecorded training videos presented by A.W. featured demonstrations of key techniques used in each treatment session. Practitioners felt that the videos improved their understanding of patient interactions and the delivery of MCT components. Practitioners further noted that they appreciated accessing the videos after the training, allowing them to re‐watch them before conducting their sessions with patients.

“Watching those deliveries, that was the most helpful because it gave you an idea of how it was sort of set up and how it works, you know, how you are sort of interacting with people.” [ID 3]

Practitioners emphasized that conducting the pilot group was essential for their learning of MCT, as it helped them solidify their understanding of MCT and gain a deeper level of knowledge and practical experience in delivering the intervention. Practitioners noted that “the penny dropped” during the pilot group delivery. Additionally, practitioners felt that the training session after the pilot group allowed them to evaluate their ability to deliver MCT further, receive additional support and guidance, address challenges encountered during the delivery, and reinforce their learning.

“I think definitely the feedback session. I thought that was really, really good because that has given us time to reflect, to look upon everything, talk about experiences … and having that [supervision session and] other people saying similar things, it kind of makes you feel, Oh, I am doing the right thing, and I think that is useful. [ID 1]

#### Training Format

The CR services involved in this study were spread across the United Kingdom, so training in MCT was delivered online. Most practitioners noted that they enjoyed the online format, felt that it was becoming standard practice, and reduced logistical issues (eg, travel).

“I think you've got to think about it logistically because if you're to have stuff done face‐to‐face, it's setting it all up, and it's the cost of it. [ID 10]

Despite this, some practitioners stated that they preferred face‐to‐face training; however, considering this, they noted that the online delivery did not adversely impact their understanding or implementation of group‐MCT.

“I liked the online [training]…I do prefer face‐to‐face in any situation, but online was fine … I still got a lot of benefits out of it, still enjoyed it, learned a lot from it.” [ID 2]

### Theme 2: Training Barriers

Three subthemes were identified when evaluating the barriers to training in group MCT: training structure, staff skepticism, and additional training needs.

#### Training Structure

The CR staff identified 2 challenges related to the online training structure: the use of role plays and the timing of receiving the training manual. They felt that the role plays could have been more effective, as when practitioners were assigned to breakout rooms, they often deviated from the intended task. To improve this in the future, they suggested that an MCT‐trained therapist should join the breakout rooms to act as a facilitator.

“ I think that in the breakout sessions, they did go astray a little bit. So, it would perhaps be useful if somebody from the research team joined those.” [ID 5]

In addition, practitioners mentioned that they would have preferred to receive the treatment manual before the training sessions. However, to promote better engagement during the training and to avoid practitioners relying on the manual while learning, the manuals were distributed after the training was completed, and the practitioners did recognize the justification for this decision.

“Possibly have the booklets beforehand or have the booklets to read during the training. But then I think, as you said in the training, sometimes if they need to look at the booklets, then they are not listening to what is going on, so it is not always that helpful.” [ID 3]

#### Staff Skepticism

At the beginning of the training, CR practitioners expressed some skepticism about the MCT techniques and whether they could serve as an effective psychological therapy. For instance, practitioners found it challenging to envision how the techniques would apply to their patients and address specific concerns they had encountered in the past. However, after completing the training and implementing MCT, therapists' perspectives changed, leading to a reduction or elimination of their initial skepticism.

“When we first started the course, I was very skeptical. I think of some of the techniques, [I] was like okay, let us see how this goes, but seeing the benefits of patients, you can see that they work really, really well…they grow and develop each week that we come in so you can really see how that helps them.” [ID 1]

“If I'm honest, I was a bit sort of skeptical about it initially at the initial training, and I suppose some of the things I was thinking about were things that, you know, some of things that the patients have come up with and having to come up with a response to that has made me look into it more and think about it more.” [ID 3]

#### Further Training Needs

Two future training needs were identified, including focusing more on challenging negative metacognitive beliefs that worrying is harmful and more information on managing challenging patients and group dynamics. Practitioners reported difficulties confronting the belief that worrying is harmful, especially in discussions about stress, a common focus in other CR work. In particular, there was confusion among practitioners concerning how stress is different from worry, and there was a recurring preconception that stress was dangerous, which seemed to work against embracing some MCT principles at the beginning of training.

“The only other thing that could be explored in more detail is the stress element to give us some more tools to be able to deal with the questions around stress and the difference between the beliefs around stress and how that is different to worrying thoughts.” [ID 2]

Although practitioners are well versed in managing CR groups, practitioners indicated that they valued further training on how to manage challenging patients and group dynamics.

“I think maybe just a bit more of it [training], I think just to prepare us for managing a group … [and] I think the managing of the dynamics.” [ID 7]

In particular, further training on how to manage the group when 1 patient dominates conversations or how to manage topics such as suicide were seen as particularly important.

“I think there's certain areas…that I found could be quite challenging, such as … how to deal with when a patient comes up and says … a friend's died recently suicide.” [ID 1]

“Chronic psychological issues like PTSD and things like that, so they were complex, but yes, perhaps more training on that would help.” [ID 2]

## DISCUSSION

This study is the first to investigate the training experiences of CR practitioners in group MCT to identify the potential barriers and facilitators of training. The practitioners reported a positive training experience, highlighting key factors that enhanced their knowledge, understanding, and application of group MCT. However, they also pointed out that barriers to training in group MCT included challenges in training structure, staff skepticism toward treatment, and future training needs.

CR practitioners emphasized that the multicomponent nature of training was crucial for improving their grasp of the intervention. Practitioners described that training videos significantly enhanced their understanding of interacting and talking effectively with patients while delivering various MCT components. They appreciated the opportunity to revisit these recordings posttraining, as this allowed them to refresh their knowledge and review specific techniques before engaging with patients in their sessions.

Moreover, the pilot delivery of group MCT was regarded as a pivotal experience for practitioners, providing them with a practical platform to apply their learning and deepen their understanding and application of MCT techniques. Several reported experiencing moments during the delivery of the pilot group where they felt that concepts “clicked” into place, reinforcing their understanding of the intervention.

Additionally, practitioners indicated that the training review session following the pilot group was immensely beneficial. This session offered them a chance to critically evaluate their performance in delivering MCT, seek further support and guidance, and discuss challenges they faced during the pilot delivery, thereby consolidating knowledge and building confidence.

The training helped practitioners feel more equipped and confident in facilitating MCT sessions with their patients. The narratives align with previous research on therapist training in EBIs for mental health, where training employing multiple methods, especially those incorporating active, behaviorally oriented learning strategies, improves practitioners' knowledge, use, and competence.^46^, ^47^

Recent studies indicate that simpler EBIs, such as measurement‐based care, can be effectively taught through online and in‐person training with similar outcomes. However, more complex EBIs, which involve multiple components (such as MCT) or require nuanced applications, may benefit more from in‐person instruction and guidance within the therapeutic context.^48^ The training for group MCT in the PATHWAY trial was conducted in person. However, due to the geographic distribution of the CR services and staff in the present study, we used an online platform for training, but we maintained the practical experience element by introducing the practice of full treatment delivery through running a pilot group followed by a summative online training session.

Most practitioners reported enjoying the online training and felt it was becoming standard practice, as it alleviated logistical challenges, such as travel. However, some expressed a preference for face‐to‐face training. Despite this preference, they noted that the online format did not negatively affect their understanding or implementation of the intervention, and they identified the delivery of the pilot group as an essential training component.

The CR staff identified 2 challenges related to online training delivery: the use of role play and the timing of receiving the training manual. Although role play is a common training technique that can enhance learning, it often creates apprehension among participants. Nevertheless, many trainees value this approach. Feedback from our trainees indicated that the use of role play could have been more effective; specifically, when practitioners were assigned to breakout rooms, they frequently deviated from the intended tasks.

Previous research has shown mixed reactions to behavioral rehearsal, with some clinicians enjoying role play and others not finding it enjoyable or useful.^49^, ^50^ Past reviews have suggested that although active training strategies, such as behavioral rehearsal (role play), lead to higher adherence and skill development after training, they do not consistently translate into greater application in practice.^46^

To improve future training sessions, CR practitioners recommended using an MCT‐trained therapist as a facilitator in the breakout rooms. This addition would help ensure that the scenarios are realistic and relevant to the trainees’ experiences, addressing a common barrier identified in previous research where role play exercises were perceived as irrelevant or frustrating.^50^

Practitioners expressed a preference for receiving the treatment manual before the training sessions. However, to encourage better engagement during the training and to prevent practitioners from relying solely on the manual while learning, the manuals were distributed after the training was completed. The practitioners understood the rationale behind this decision. Previous research has shown that merely reading treatment manuals does not lead to significant knowledge acquisition or skill development.^51^ Therefore, the manual should be viewed as a supplementary resource that should not detract from active learning during training.

Previous research has indicated that clinicians often doubt the effectiveness of new treatment methods.^39^ This aligns with our findings, which show that at the beginning of their training, CR practitioners were skeptical about the efficacy of MCT techniques as a form of psychological therapy within the CR context. Some practitioners struggled to envision how the techniques could be applied to their patients or how they could address specific concerns they had encountered in the past. However, after completing training and implementing MCT with their patients, the therapists' perspectives shifted, leading to a reduction or elimination of their initial skepticism. Two key facilitators of change in staff skepticism were the delivery of a pilot group and a follow‐up training session afterwards. These allowed practitioners to directly experience therapy delivery and provided evidence of contextual relevance and positive outcomes. However, proactively eliciting and discussing expectations and skepticism about therapy in the training sessions may be helpful to facilitate engagement in the early phases of training in the future. In addition, future training could incorporate patient feedback that may help to allay skepticism in the initial training sessions.

CR practitioners identified future training needs, including addressing negative metacognitive beliefs that worrying is harmful and reconciling MCT with existing unhelpful beliefs that practitioners held about stress. Previous research found that patients often view stress and worry as harmful and dismiss current stress management and relaxation techniques offered during CR sessions as superficial and hard to implement in practice.^52^ Additionally, CR practitioners have expressed hesitance in discussing stress and the effectiveness of relaxation methods, believing these do not adequately address the core issues of anxiety and worry.^26^

MCT emphasizes that stress and worry alone are not harmful but can lead to unhealthy behaviors that increase patients’ risk of heart disease. This message aligns with the British Heart Foundation's guidance,^53^ yet many patients believe that stress is damaging, a view that is often and unhelpfully reinforced by medical professionals. Therefore, it is essential that MCT training effectively covers the theory regarding the harmless nature of worry and equips CR practitioners with the knowledge and ability to discuss and challenge patients' beliefs about stress as well as challenging their own unhelpful beliefs.

CR practitioners identified the need for further guidance in managing more challenging patient interactions, navigating group dynamics, and discussing sensitive topics such as suicide. This is especially important for staff members who may not have had previous mental health training. In group settings, some participants can dominate discussions whereas others may be reluctant to engage, hindering the group's progress. Staff training is essential to facilitate effective group therapy, encourage positive participation, and address conflicts constructively.

## STRENGTHS AND LIMITATIONS

The current qualitative study is the first to explore practitioners' perspectives following training in group MCT within CR. All interviewed practitioners consented to both the training and its delivery. Their interest in mental health and desire to enhance psychological support within CR services may have influenced their training experiences.

This study has several strengths. The practitioners interviewed are representative of the larger CR workforce. Furthermore, we included practitioners from various services with diverse roles and experience levels, which provides a broad range of perspectives.

The involvement of multiple researchers from different backgrounds in data analysis helped reduce individual bias and improved the quality of our interpretations. However, several weaknesses exist, including a small sample size and an underrepresentation of men in the sample, which may affect the themes identified.

Despite the limitations, the results support several recommendations for implementing MCT in CR settings. First, training was positively received by a workforce with limited prior knowledge of complex EBIs, supporting the transferability of MCT to clinical settings. Group MCT may be well suited to a range of CR programs beyond the UK National Health Service context given that group MCT focuses on transdiagnostic mechanisms that underlie common mental health symptoms and can be delivered by non‐mental health professionals, making it well suited to global programs, where a range of different professions; nurses, dietitians, and physiotherapists are the most common providers.^54^ Second, brief online training supported by videotape illustrations of techniques and followed by practice of running a course of therapy was viewed as facilitative in developing an effective understanding and confidence in MCT skills use. Within each CR service, it would seem to be advantageous for practitioners to engage in training in MCT to enhance psychological knowledge and skills and support the unmet psychological needs of patients.

## CONCLUSIONS

The results of the present study identify essential facilitators and barriers to a structured MCT training program delivered to CR practitioners in the National Health Service. The information gained from the analysis and emergent themes can help to refine training, pointing to particular use of facilitator supported role plays, modules supporting the management of challenging patient dynamics, chronic psychological symptoms (eg trauma), and sensitive topics. The findings support subsequent implementation and MCT rollout in the CR pathway aimed to improve the mental health of patients with cardiovascular disease and increase the knowledge and skill sets of practitioners.

## Sources of Funding

This article presents independent research funded by the National Institute for Health and Care Research under its Programme Development Grants (National Institute for Health and Care Research: 202956). The views expressed are those of the author(s) and not necessarily those of the National Institute for Health and Care Research or the Department of Health.

## Disclosure

Professor Wells is the director of the MCT Institute and developer of MCT. He has authored books on cognitive behavior therapy and MCT. The other authors report no conflicts.

## Supporting information

COREQ Checklist
